# An app for apples: Citizen-led mapping of fire blight in Central Asia

**DOI:** 10.1007/s42161-023-01406-0

**Published:** 2023-05-30

**Authors:** Mirjam Kurz, Ormon Sultangaziev, David Szalatnay, Ishenbai Sodonbekov, Dinara A. Naizabayeva, Muqaddas Milikbekova, Solimshoh Akbarsho, Saykal Bobushova, Tinatin Doolotkeldieva, Fabio Rezzonico, Theo H. M. Smits

**Affiliations:** 1 Environmental Genomics and Systems Biology Research Group, Institute for Environment and Natural Resources, Zürich University for Applied Sciences (ZHAW), Wädenswil, Switzerland; 2 Fauna & Flora International Kyrgyzstan, Bishkek, Kyrgyz Republic; 3 Strickhof Competence Centre for Agriculture and Food Management, Winterthur, Switzerland; 4 National Academy of Sciences of Kyrgyz Republic, Bishkek, Kyrgyzstan; 5 Tethys Scientific Society, Almaty, Kazakhstan; 6 M.A. Aitkhozhin Institute of Molecular Biology and Biochemistry, Almaty, Kazakhstan; 7 Fauna & Flora International Tajikistan, Dushanbe, Tajikistan; 8 Kyrgyz-Turkish Manas University, Bishkek, Kyrgyzstan

**Keywords:** *Malus*, *Pyrus*, Citizen science, Mobile device, Android

## Abstract

Fire blight, caused by the bacterial pathogen *Erwinia amylovora*, is a severe bacterial disease of apple and pear that can quickly destroy whole plants. In the last decade, it was also detected in Central Asia, where wild pomaceous fruit plants represent the dominant species in mid-altitude forests and constitute a critical foundation for the entire ecosystem. Efficiently informing farmers, forestry services and private persons about the instances and dangers of fire blight, the correct way to recognize the symptoms, and the methods of disease control is thus of paramount importance in a vast and fragmented natural landscape like the one characterizing countries like Kyrgyzstan, Kazakhstan and Tajikistan. For that purpose, we have developed an app for smartphones and mobile devices that can inform stakeholders about fire blight, simultaneously allowing a citizen science approach for mapping the spread of the disease in Central Asia. The app is available in the three national languages as well as in Russian, English, and German, and can easily be adapted to new countries, languages or even diseases.

Fire blight, caused by the bacterium *Erwinia amylovora*, has been spreading throughout the world for over a century, causing great economic losses in pome fruit production. First detected on apple trees in the Hudson Valley (State of New York) in the 1790s, fire blight has moved to New Zealand in the 1920s. In 1958, it was then detected in the United Kingdom, before gradually spreading to all European countries, to North Africa and to the Middle East in the second part of the 20th century (Bonn and van der Zwet 2000). In the last 20 years, fire blight has been expanding eastward out of Europe and the Mediterranean area towards Russia, Caucasus and Central Asia (Drenova et al. 2013; Doolotkeldieva and Bobusheva 2016).

The threat represented by fire blight in Central Asia requires particular consideration, since domesticated apple and pear species originate from this area (Maltseva et al. in press). Still today, wild apple species represent the dominant species in mid-altitude forests and constitute a critical foundation for entire ecosystems as well as precious reservoir of genetic diversity for pomaceous species. At the same time, the production of apples and pears is an important source of income for the human population in the area. The disease was first detected in the area in 2008 in Kyrgyz and Kazakh orchards (Drenova et al. 2013; Doolotkeldieva and Bobusheva 2016). Because fire blight has only recently emerged in the region, the knowledge of citizens and fruit growers but also of plant disease inspectors on how to recognize and control the disease is still relatively limited.

An international team of scientists from Switzerland, Kyrgyzstan and Kazakhstan and non-governmental organizations (NGOs) operating in Central Asia is collaborating within the Swiss National Research Foundation r4d project “Preservation of Central Asian fruit tree forest ecosystems, pome fruit varieties and germplasm from the recent epidemics caused by the invasive bacterial pathogen *Erwinia amylovora* (fire blight)” on disseminating information about the disease in Central Asia. Currently, this is still based on establishing direct contacts with the local population and handing out illustrated information brochures that were translated to the respective languages. However, this is unpractical considering the difficulties of travelling in such an extensive and fragmented territory. Additional information channels were deemed useful. Since most people have access to the internet via mobile devices, the development of a dedicated app could greatly enhance the transfer of knowledge directly where it is needed.

Thus, the aim of this work was to develop and distribute an app for smartphones and tablets on the topic of fire blight with two main objectives: informing local populations in Central Asia about fire blight and its symptoms, while also allowing to monitor the spread of the disease using a citizen science approach. Initial target users were specialized users, for example local authorities, scientists or foresters, with the aim to extend to all interested citizens. The content was to be delivered by means of easy communication for users which do not have a scientific background.

The developed app, called “Fire Blight”, should provide visual and textual information on fire blight, such as symptoms of the disease, possible misidentification sources and management measures. In addition, a tool to monitor the spread of fire blight was to be included (Fig. 1). To meet these goals, the app was organized in five main chapters (Fig. 2A), with four of them focusing on knowledge transfer and one on reporting of infections to allow monitoring of the spread of fire blight in Central Asia.

**Fig. 1:**
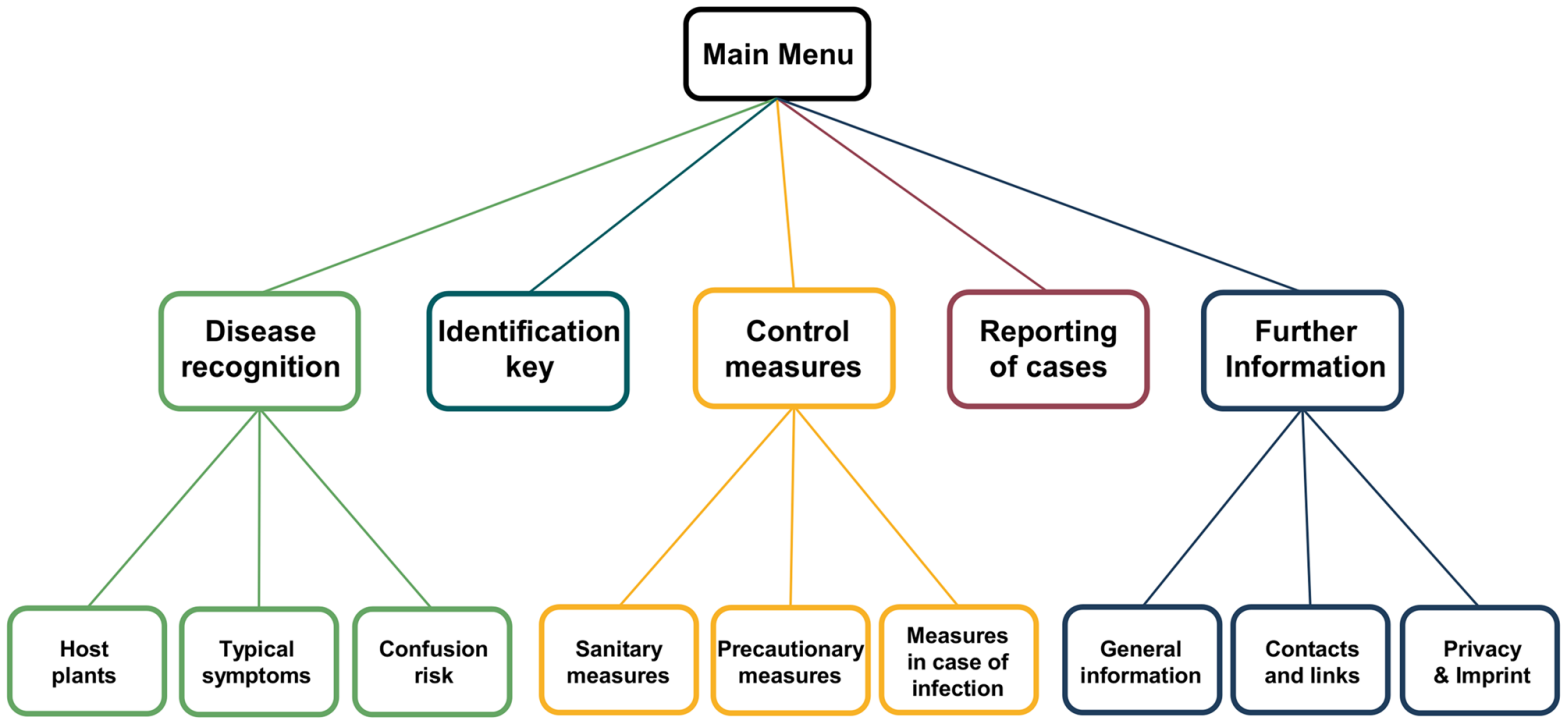
Structure of the fire blight app. After entry in the app (main menu), the user can choose the topic of interest, with subcategories for some of the topics

**Fig. 2:**
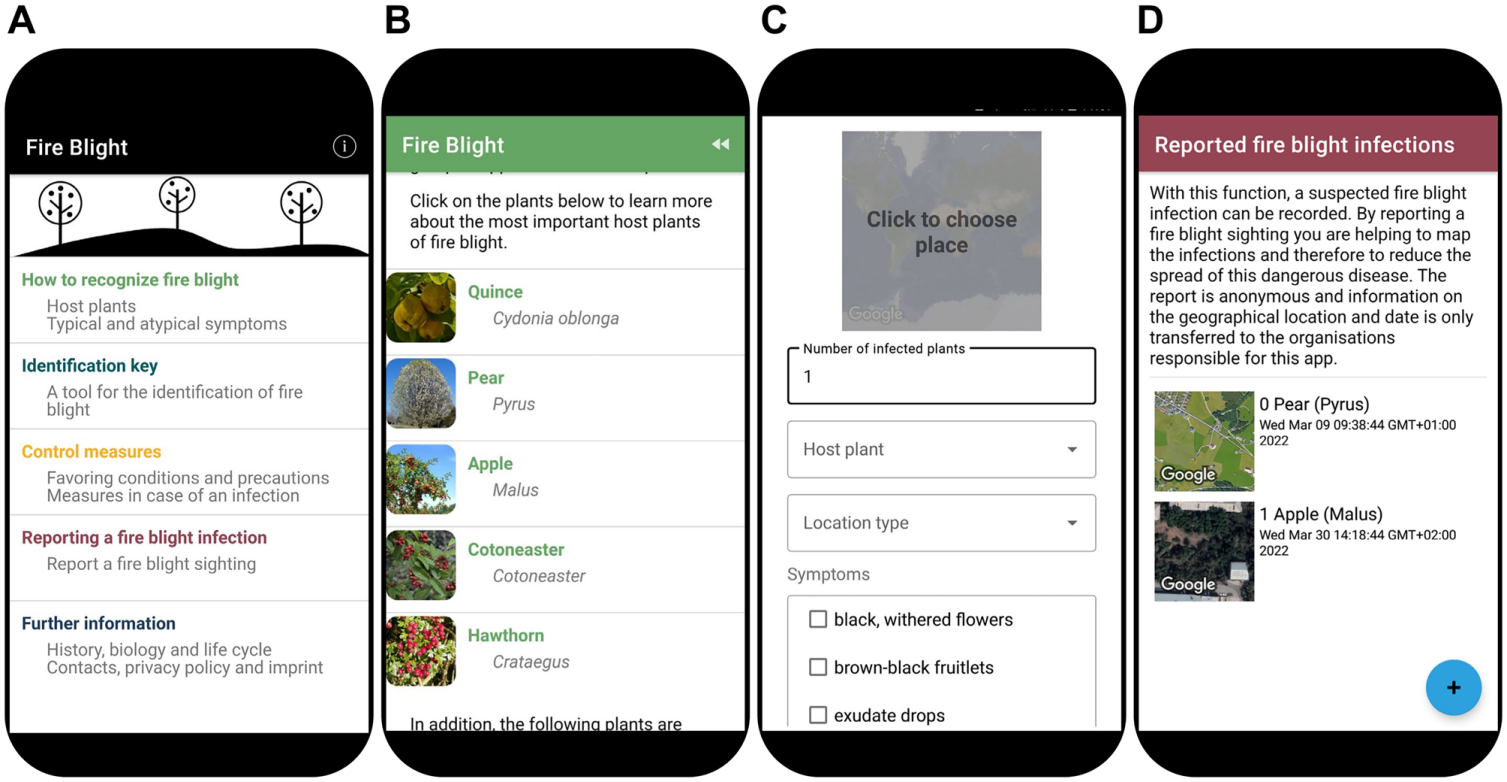
Screenshots from the app. **A**: Main menu with the five main sections. **B**: Selection screen for information on fire blight host plants. **C**: Entry screen for reporting a fire blight case. **D**: Final screen after reporting with already reported cases by the user

The first section on disease recognition facilitates fire blight diagnostics for a layman. Since advisors were faced with many time-consuming inquiries about fire blight symptoms on non-host plants, an extensive list of known host plants for fire blight was presented. For the economically most important host plants, i.e., apple, pear, quince, hawthorn, and cotoneaster, more specific information and pictures of healthy and infected plants were included (Fig. 2B). Furthermore, descriptions of typical symptoms of fire blight on different plant parts were supplemented with many pictures. Because other diseases or abiotic factors such as mechanical damage can result in similar symptoms to fire blight on host plants, known misidentification sources were listed and discriminating signs were described.

During the development of the content, it quickly emerged that it was challenging to describe the various symptoms of fire blight and of the diseases with which it can be confounded at a glance. Therefore, an identification key based on simple yes/no questions and complemented with exemplary pictures was specifically developed for the app. By answering between two and 13 questions, the user can determine whether a diseased plant is most probably infected by fire blight or if the observed symptoms are caused by a different disease.

The third part of the app covers issues connected to management of fire blight infections in orchards. Since it is of essential importance to stop the spread of the disease in case of an outbreak (Johnson and Stockwell 1998), sanitation measures are presented. Measures such as disinfection of tools or burning of infected material play an important role in this process (Gusberti et al. 2015). Precautionary measures on how to protect plants from infections are also included. Additionally, information on how to proceed in case a fire blight infection is predicted and how to cut back and destroy infected material can be found in this part.

The app uses a citizen science approach for data collection to monitor the spread of fire blight in Central Asia. Layman and experts can report potential fire blight infections in the app via the “reporting of cases” part (Fig. 2C, D). Questions on the number, species and location of infected plants, the observed symptoms and confirmation method (either visual or with a laboratory or on-field test) must be answered. The entries are treated anonymously, and data sorted by country is stored on a server in Switzerland. Utilizing the gathered data, a more targeted field monitoring approach can be implemented, thus improving the overall response to a fire blight outbreak. This will result in a clearer picture of the risk for fire blight around the endangered apple and pear species in the forests of Central Asia, while allowing the app users to better protect their orchards and gardens.

The last section of the app contains basic information on the disease, such as the history of fire blight and the life cycle of the pathogen *E. amylovora* (Van der Zwet et al. 2016). Information on the project team, local contacts, and links to further information about the disease is included as well. The exact content of the app was developed based on specific needs of the target countries (for example varying host species) and is mostly based on fact sheets produced earlier by Agroscope in Switzerland (Duffy et al. 2005).

In June 2022, a preliminary version was tested by the r4d project consortium during a field trip in Kyrgyzstan. Positive sightings, confirmed by onsite use of the Agristrip Ea immunoassay (Braun-Kiewnick et al. 2011) and recent isolation of bacteria from samples in the lab were entered in the system already as test cases. This test run showed the usefulness of the app in the field, while smaller adaptations were made. Since September 28th, 2022, the fire blight app is available on Google Play for Android users in six languages (English, German, Russian, Kyrgyz, Kazakh and Tajik). The choice to first develop the Android version was deliberate, as the majority of users in Central Asia utilizes this operative system on their mobile phone. As of April 12, 2023, the app “Fire Blight” has been downloaded a total of 153 times; 72 of which occurred in Kyrgyzstan. A version for Apple iOS is currently in development.

The application was initially introduced to local governments, plant protection department employees, forestry workers, and local residents in Kyrgyzstan. According to preliminary observations, the general perception is that the application will facilitate the detection of the disease allowing protective work to be carried out in a timely manner. One plant protection office stated that, in the future, they will help local farmers with the distribution of the application. It is now planned to conduct series of training sessions with rangers, local people, and relevant stakeholders on how to use the fire blight app, and to manage their orchards and leased forest plots at the project sites. Additionally, information about the fire blight app is disseminated to a wider public by using radio, TV, and social media platforms in the region.

Currently, the collected case data must be worked up manually for each country, and cannot be displayed immediately in the app. One possibility is the coupling of the geolocation with a map of the region, so that users immediately can see where fire blight infections have taken place recently. This information is of importance for the regulatory agencies, to define local and regional measures against the disease. Although currently developed and adapted to Central Asia, it is possible to translate the content easily for application of the app in other countries as well.

To optimize the fire blight app further, the possibilities of symptom identification from pictures by automatic image recognition should be investigated. While the current identification key is a solid solution for disease diagnostics, there are promising developments in automatic image recognition for plant diseases (Jarolmasjed et al. 2019; Kang et al. 2020). Using this approach, image recognition would be a valuable complement to the determination key and could further improve the early detection of fire blight for laymen.

## Data Availability

All data on the study is included in the manuscript. Further information on the app can be obtained from the authors.
